# Belantamab Mafodotin Plus Proteasome Inhibition Efficacy Versus Comparators in Early Relapsed Myeloma: A Systematic Review and Network Meta‐Analysis

**DOI:** 10.1002/ajh.27661

**Published:** 2025-03-27

**Authors:** Joshua Richter, Ajay Nooka, Paula Rodríguez‐Otero, Fredrik Schjesvold, Eirini Katodritou, Emily Combe, Marianne Scott, Leanne Cooper, Indeg Sly, Nick Ballew, Jacopo Bitetti, Natalie Boytsov, Molly Purser, Simon McNamara

**Affiliations:** ^1^ Mount Sinai New York New York USA; ^2^ Winship Cancer Institute of Emory University Atlanta Georgia USA; ^3^ Cancer Center Clinica Universidad de Navarra Pamplona Spain; ^4^ Oslo Myeloma Center, Department of Hematology Oslo University Hospital Oslo Norway; ^5^ Theagenio Cancer Hospital Thessaloniki Greece; ^6^ FIECON London UK; ^7^ GSK Upper Providence Pennsylvania USA; ^8^ GSK Zug Switzerland; ^9^ GSK, Stevenage Hertfordshire UK

**Keywords:** belantamab mafodotin combination, efficacy, meta‐analysis, relapsed/refractory multiple myeloma

## Abstract

In the Phase 3 DREAMM‐7 study of patients with relapsed/refractory multiple myeloma (RRMM) who received ≥ 1 prior therapy, belantamab mafodotin plus bortezomib and dexamethasone (BVd) demonstrated a progression‐free survival (PFS) benefit versus daratumumab plus bortezomib and dexamethasone (DVd). This study aimed to indirectly compare the efficacy of BVd against alternative regimens in this patient population. A systematic literature review (SLR; December 2021–February 4, 2024) was performed to identify relevant efficacy data. Studies were selected based on the Population‐Intervention‐Comparators‐Outcomes‐Study design framework criteria and independently reviewed for inclusion in the network meta‐analysis (NMA) if they had a connection to DREAMM‐7 (approved in the US or EU, or likely to be a future DREAMM‐7 comparator). Each trial had a common comparator arm, allowing for a connected network between the trials and linkage by shared treatments. The primary analysis was PFS in the intent‐to‐treat population from each study, and secondary analyses examined other endpoints. All endpoints were also evaluated in subgroups by lenalidomide‐exposure, ‐refractoriness, and other patient characteristics. The SLR identified 12 comparator studies comprising 12 comparator regimens (each contained a proteasome inhibitor [bortezomib or carfilzomib] plus dexamethasone), all of which were included in the NMA with the DREAMM‐7 study. BVd improved PFS versus all comparators, including daratumumab plus carfilzomib and dexamethasone, isatuximab plus carfilzomib and dexamethasone, and DVd. Overall survival was also improved by belantamab mafodotin plus bortezomib and dexamethasone over the other regimens. This study provides compelling evidence for belantamab mafodotin, plus bortezomib and dexamethasone, in early lines of treatment for RRMM.

## 1 Introduction

Multiple myeloma (MM) is a hard‐to‐treat hematologic malignancy associated with poor prognosis and quality of life [1, 2]. Belantamab mafodotin is an antibody–drug conjugate (ADC) targeting B‐cell maturation antigen with a multi‐modal mechanism of action including direct, ADC‐mediated cytotoxicity, antibody‐dependent cellular cytotoxicity and phagocytosis, and induced immunogenic cell death [3].

Due to high overall response rates (ORR) reported for belantamab mafodotin monotherapy in DREAMM‐1, ‐2 and ‐3 [4, 5, 6], and preclinical synergy observed between belantamab mafodotin with pomalidomide and with bortezomib [7], additional phase 3 trials, DREAMM‐7 [8] and DREAMM‐8 [9], investigated belantamab mafodotin in different combination regimens versus standard of care. DREAMM‐7 (NCT04246047) evaluated belantamab mafodotin + bortezomib (V) and dexamethasone (d; BVd) versus daratumumab + Vd (DVd) in patients with relapsed/refractory MM (RRMM) who had ≥ 1 prior line of therapy (LOT) and demonstrated a significant benefit in progression‐free survival (PFS) in favor of BVd (hazard ratio [HR] 0.41, 95% confidence interval [CI] 0.31–0.53, *p* < 0.001): median PFS (95% CI) was 36.6 (28.4–not reached [NR]) versus 13.4 (11.1–17.5) months for BVd versus DVd, respectively. At the primary analysis (median follow‐up: 28.2 months), median overall survival (OS) was not reached in either arm (HR 0.57, 95% CI 0.40–0.80) with follow‐up ongoing. The ORR (95% CI) was 83% (77–87) for BVd and 71% (65–77) for DVd with a doubling of complete response rate or better (35% [29–41] vs. 17% [13–22], respectively) [8].

Multiple combination regimens are available for MM/RRMM treatment, and regimen choice is complex and multifactorial [10]. Patients are becoming exposed to commonly used drugs in early LOTs, including the immunomodulatory drug lenalidomide (R) [11, 12, 13] which is a standard in first‐line treatment based on positive outcomes from the FIRST study (Rd vs. R vs. melphalan, prednisone, and thalidomide) [14] and more recent studies, MAIA (DRd vs. Rd) [15] and PERSEUS [16] (D‐VRd vs. [VRd]). The outcomes of these studies are anticipated to increase the likelihood of patients becoming exposed to and refractory to commonly used drugs, including lenalidomide and daratumumab, in earlier stages of disease [12, 17], thereby increasing the need for subsequent, alternative regimens with different mechanisms of action to prolong survival outcomes.

Without head‐to‐head studies (other than the comparison to DVd in DREAMM‐7) [8], indirect treatment comparisons are valuable to evaluate the relative efficacy of BVd against alternative regimens for patients with RRMM who had received at least one prior LOT. Our study indirectly examined BVd efficacy in patients with RRMM relative to other regimens and considered subpopulations with unmet clinical needs.

## 2 Methods

This systematic literature review (SLR) and network meta‐analysis (NMA) is reported in accordance with the Preferred Reporting Items for Systematic Reviews and Meta‐analyses reporting guideline extension statement for systematic reviews, incorporating NMA for healthcare interventions [18].

### 2.1 Literature Search and Study Selection

A SLR was performed in December 2021 and the last search update was carried out on February 4, 2024, to identify all relevant efficacy data for regimens used in the management of RRMM for patients who had received at least 1 prior LOT (Figure S1A). The full electronic search strategy is shown in Table S1. Studies were screened in the title/abstract and full‐text steps according to the Population‐Intervention‐Comparators‐Outcomes‐Study design framework criteria [19] (listed in Table S2). Titles and abstracts identified by the search strategy were independently assessed by two reviewers. Any discrepancies between the two reviewers were resolved by a third senior reviewer. Risk of bias was conducted in the feasibility assessment using the Revised Cochrane risk of bias tool for individually randomized, parallel‐group trials (RoB2.0) [20]. Heterogeneity in patient population characteristics between the studies was also assessed.

### 2.2 NMA Study Design

Studies identified in the SLR were included in the NMA if they had a connection to the DREAMM‐7 study [8] and these included regimens that were approved by the U.S. Food and Drug Administration or European Medicines Agency or were likely to be a future Health Technology Assessment comparator to the DREAMM‐7 study. This study used an anchored, indirect/mixed treatment comparison design, where there was a common comparator arm in each trial, allowing a connected network between the trials to be formed. Trials were linked together by the treatment(s) they shared to form connected networks of evidence. Trials/regimens that were not part of the connected evidence networks were excluded from the NMA.

An NMA feasibility assessment was carried out in two stages:

1. Confirmation that a network of studies could be constructed for each outcome (through evaluation of qualitative descriptions) such that an indirect assessment of study outcomes could be carried out.
2. Assessment of clinical and methodological similarity among network studies.

### 2.3 Data Analyses

The primary analysis examined PFS in the intent‐to‐treat (ITT) population as reported in each study. Where data were available, secondary analyses examined OS and ORR in the ITT population, as well as PFS and all other endpoints in the lenalidomide‐exposed and lenalidomide‐refractory populations. Based on available data, subgroup network analyses were also carried out to explore outcomes according to patient characteristics that have the potential to impact the effect of treatment, such as high cytogenetic risk and the number of prior LOTs.

Fixed‐effects and random‐effects models were run following the NMA using National Institute of Health and Care Excellence Decision Support Unit recommended methodology [21]. For the fixed‐effects model, a normal likelihood and the identity link function were used for time‐to‐event outcomes. The random‐effects models accounted for potential heterogeneity in follow‐up time and dosing regimens between studies included in the NMA, and based on the available evidence, a set of priors for between‐study variance in studies with binomial outcomes was used to inform between‐study standard deviations for time‐to‐event outcomes. HRs and 95% credible intervals (CrI) were computed for BVd relative to each comparator, and binomial likelihood and the logit link function were used for binomial outcomes; odds ratios (OR) and 95% CrIs were computed.

The statistical analyses were run using the multiNMA package version 0.7.0 and R version 4.2.1 or above via Rstudio to perform the analysis. An HR less than 1 indicated BVd was more effective than the comparator and an OR greater than 1 indicated BVd was associated with a greater likelihood of the event (ORR) than the comparator; for these HR/ORs, a CrI excluding 1.0 indicated a high probability that the treatment effect is consistently in favor of BVd.

Statistical fit for each model was assessed by deviance information criterion, the median residual deviance statistic, and the effective number of parameters as a measure of model complexity.

## 3 Results

### 3.1 Study Selection and Network Geometry

In total, 12 relevant comparator studies were identified in the SLR (Figure S1B) [22, 23, 24, 25, 26, 27, 28, 29, 30, 31, 32, 33, 34, 35, 36, 37, 38, 39, 40]; one publication, which was not captured within the SLR, was added afterwards as it reports updated PFS and OS data for the OPTIMISMM trial [41]. The number of patients per regimen and patient subgroup for each trial are presented in Table 1. Heterogeneity assessment determined that baseline characteristics between the studies were generally similar (Table S3). The overall risk of bias was heterogeneous among included studies, but no studies were regarded as outliers. Based on the risk of bias assessment, it was not recommended to exclude any studies. The network graph of treatment comparisons is presented in Figure S2.

**TABLE 1:** Study selection by treatment regimen.

Acronym	Total number of patients per regimen, *N*	Trial	ITT, *n*	Len‐exposed, *n*	Len‐refractory, *n*	1 Prior line, *n*	High‐risk cytogenetic, *n*
BVd	243	DREAMM‐7 [8]	243	127	79	125	67
DVd	643	DREAMM‐7	251	130	87	125	69
		LEPUS [22, 23]	141	NA	NA	41	46
		CASTOR [24, 25, 42, 43]	251	89	60	122	40
Vd	1766	LEPUS	70	NA	NA	19	27
		CASTOR	247	120	81	113	35
		NCT00813150 [26]	43	NA	NA	NA	NA
		OPTIMISMM [26, 27, 41]	278	278	191	115	49
		BOSTON [28, 40, 44]	207	77	53	99	71
		PANORAMA‐1 [29, 30]	381	NA	NA	174	NA
		NCT01478048 [31]	75	NA	NA	51	NA
		ENDEAVOR [32, 33, 34, 45]	465	177	122	229	113
CyVd	47	NCT00813150	47	NA	NA	NA	NA
EVd	77	NCT01478048	77	NA	NA	50	NA
hKd	979	ENDEAVOR	464	177	113	231	97
		CANDOR [35, 36, 46]	154	74	55	67	26
		ARROW [37]	238	194	170	NA	47
		IKEMA [38, 47]	123	59	42	55	31
PanoVd	387	PANORAMA‐1	387	NA	NA	178	NA
PVd	281	OPTIMISMM	281	281	200	111	61
SVd	195	BOSTON	195	77	53	99	70
DKd	312	CANDOR	312	123	99	133	48
IsaKd	179	IKEMA	179	72	57	80	42
Kd	340	ARROW	240	207	186	NA	34
		GEM‐KyCyDex [39]	100	NA	46	NA	28
CyKd	94	GEM‐KyCyDex	97	NA	43	NA	24

Abbreviations: BVd, belantamab mafodotin + bortezomib + dexamethasone; CyKd, cyclophosphamide + carfilzomib + dexamethasone; CyVd, cyclophosphamide + bortezomib + dexamethasone; DKd, daratumumab + carfilzomib + dexamethasone; DVd, daratumumab + bortezomib + dexamethasone; EVd, elotuzumab + bortezomib + dexamethasone; hKd, high‐dose carfilzomib + dexamethasone; IsaKd, isatuximab + carfilzomib + dexamethasone; ITT, intent‐to‐treat; Kd, carfilzomib + dexamethasone; Len, lenalidomide; NA, not available; PanoVd, panobinostat + bortezomib + dexamethasone; PVd, pomalidomide + bortezomib + dexamethasone; SVd, selinexor + bortezomib + dexamethasone; Vd, bortezomib + dexamethasone.

### 3.2 Intent‐To‐Treat Population Outcomes

A total of 13 studies (including DREAMM‐7) [8] and 12 comparator regimens [22, 23, 24, 25, 26, 27, 28, 29, 30, 31, 32, 33, 34, 35, 36, 37, 38, 39, 40, 41] were included in the connected network of evidence for PFS, OS, and ORR. All 12 comparator regimens included a proteasome inhibitor (PI) in combination with dexamethasone, either bortezomib with dexamethasone (Vd) or carfilzomib with dexamethasone (Kd). Of note, to facilitate inclusion of the ARROW and GEM_KyCyDex studies in the network, equivalence of the twice‐weekly 27 mg/m^2^ Kd regimen in the ARROW study and the 56 mg/m^2^ Kd regimen in the ENDEAVOR and CANDOR studies was assumed.

**FIGURE 1:**
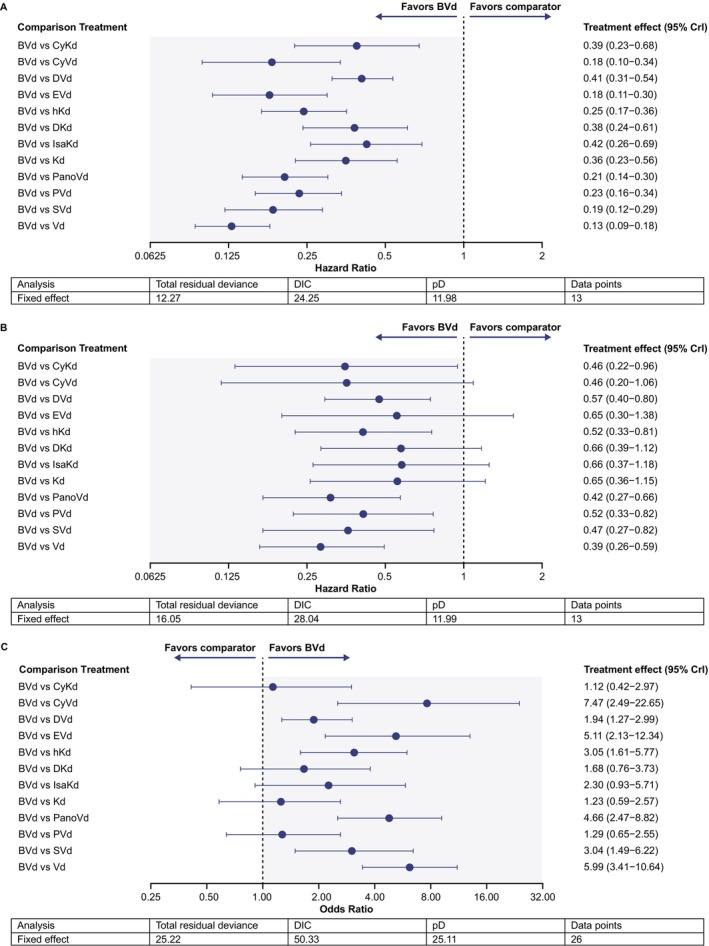
Fixed‐effect BVd treatment comparisons for intent‐to‐treat population by PFS (A), OS (B) and ORR (C). BVd, belantamab mafodotin + bortezomib + dexamethasone; CrI, credible interval; CyKd, cyclophosphamide + carfilzomib + dexamethasone; CyVd, cyclophosphamide + bortezomib + dexamethasone; DIC, deviance information criterion; DKd, daratumumab + carfilzomib + dexamethasone; DVd, daratumumab + bortezomib + dexamethasone; EVd, elotuzumab + bortezomib + dexamethasone; hKd, high‐dose carfilzomib + dexamethasone; IsaKd, isatuximab + carfilzomib + dexamethasone; Kd, carfilzomib + dexamethasone; ORR, overall response rate; OS, overall survival; PanoVd, panobinostat + bortezomib + dexamethasone; pD, effective number of parameters as a measure of model complexity; PFS, progression‐free survival; PVd, pomalidomide + bortezomib + dexamethasone; SVd, selinexor + bortezomib + dexamethasone; Vd, bortezomib + dexamethasone. [Color figure can be viewed at wileyonlinelibrary.com]

BVd improved PFS versus all comparators (Figure 1A); all CrIs were below 1, indicating a high probability that the treatment effect consistently favored BVd. Comparisons against regimens of interest included BVd versus daratumumab + Kd (DKd, PFS HRs [95% CrI]: 0.38 [0.24–0.61]), BVd versus isatuximab + Kd (IsaKd, 0.42 [0.26–0.69]), and BVd versus DVd (0.41 [0.31–0.54]).

BVd extended OS versus Vd, cyclophosphamide + Kd (CyKd), DVd, high‐dose Kd (hKd), panobinostat + Vd (PanoVd), PVd, and selinexor + Vd (SVd); CrIs were below 1. Although all results showed numerical improvement, some CrIs spanned 1, including for Kd, IsaKd, cyclophosphamide + Vd (CyVd), elotuzumab + Vd (EVd), and DKd (Figure 1B) indicating uncertainty about the treatment effect. HRs (95% CrI) reported for comparators of interest versus BVd were DKd: 0.66 (0.39–1.12), IsaKd: 0.66 (0.37–1.18), and DVd: 0.57 (0.40–0.80).

BVd demonstrated numerically superior ORR against all comparators except CyKd, DKd, IsaKd, Kd, and PVd, where ORR CrIs spanned 1 (Figure 1C). ORs (95% CrI) ranged from 1.12 (0.42–2.97) versus CyKd to 7.47 (2.49–22.65) versus CyVd. ORs (95% CrI) reported for regimens of interest versus BVd were 1.68 (0.76–3.73), 2.30 (0.93–5.71), and 1.94 (1.27–2.99) for DKd, IsaKd, and DVd, respectively.

### 3.3 Lenalidomide‐Exposed and ‐Refractory Subpopulation Outcomes

For both the lenalidomide‐exposed and ‐refractory subpopulations, NMA of OS was not feasible due to a lack of reported data to form a connected network.

**FIGURE 2:**
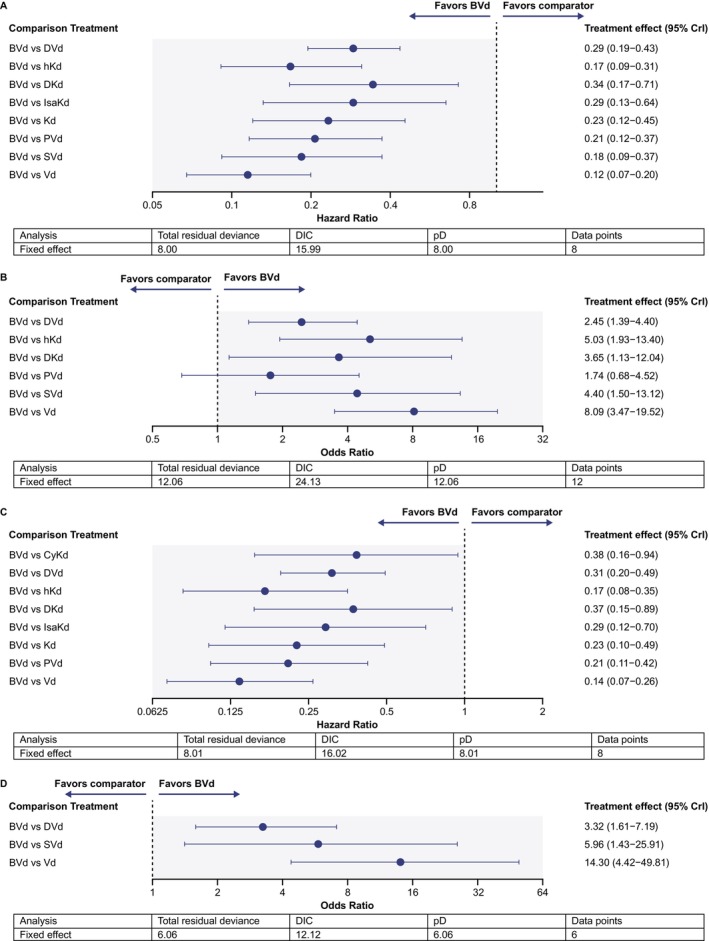
Fixed‐effect BVd treatment comparisons for lenalidomide‐exposed subpopulation by PFS (A) and ORR (B) and for lenalidomide‐refractory subpopulation by PFS (C) and ORR (D). BVd, belantamab mafodotin + bortezomib + dexamethasone; CrI, credible interval; CyKd, cyclophosphamide + carfilzomib + dexamethasone; DIC, deviance information criterion; DKd, daratumumab + carfilzomib + dexamethasone; DVd, daratumumab + bortezomib + dexamethasone; hKd, high‐dose carfilzomib + dexamethasone; IsaKd, isatuximab + carfilzomib + dexamethasone; Kd, carfilzomib + dexamethasone; ORR, overall response rate; pD, effective number of parameters as a measure of model complexity; PFS, progression‐free survival; PVd, pomalidomide + bortezomib + dexamethasone; SVd, selinexor + bortezomib + dexamethasone; Vd, bortezomib + dexamethasone. [Color figure can be viewed at wileyonlinelibrary.com]

For PFS in the lenalidomide‐exposed subpopulation, a total of 8 studies (including DREAMM‐7) [8] and 8 comparator regimens were included in the connected network of evidence [27, 32, 36, 37, 40, 41, 42, 45, 47, 48]. BVd resulted in a longer PFS compared with all comparators in the network (Figure 2A; HR [95% CrI] range: 0.12 [0.07–0.20]– 0.34 [0.17–0.71]), including comparator regimens of interest, DKd (0.34 [0.17–0.71]), IsaKd (0.29 [0.13–0.64]), and DVd (0.29 [0.19–0.43]). BVd improved ORR versus DVd, hKd, DKd, SVd, and Vd (overall OR [95% CrI] range: 1.74 [0.68–4.52]– 8.09 [3.47–19.52]), and with a lesser degree of certainty, versus PVd, as indicated by CrI spanning 1 (Figure 2B).

For PFS in the lenalidomide‐refractory subpopulation, a total of 8 studies (including DREAMM‐7) [8] and 8 comparator regimens were included in the connected network of evidence [24, 27, 32, 36, 37, 38, 39, 44, 46, 48]. BVd improved PFS versus all comparators, with all CrIs reported below 1 (Figure 2C; HR [95% CrI] range: 0.14 [0.07–0.26] – 0.38 [0.16–0.94]). HRs (95% CrI) reported for comparator regimens of interest versus BVd were 0.37 (0.15–0.89), 0.29 (0.12–0.70), and 0.31 (0.20–0.49) for DKd, IsaKd, and DVd, respectively. The NMA of ORR showed that BVd improved ORR versus all comparators in the network (OR [95% CrI] range: 3.32 [1.61–7.19] – 14.30 [4.42–49.81]). This NMA only allowed comparison to DVd, SVd, and Vd based on data from DREAMM‐7, BOSTON, and CASTOR trials, due to a lack of reported data and the absence of connecting studies which would have allowed for the inclusion of additional trials into the network (Figure 2D).

### 3.4 1 Prior Line Subpopulation Outcomes

The NMAs conducted for the subgroup analyses by 1 prior line and high‐risk cytogenetics subpopulations only considered PFS to assess the potential for bias resulting from differences in treatment effect modifiers in the studies with data available for these populations.

**FIGURE 3:**
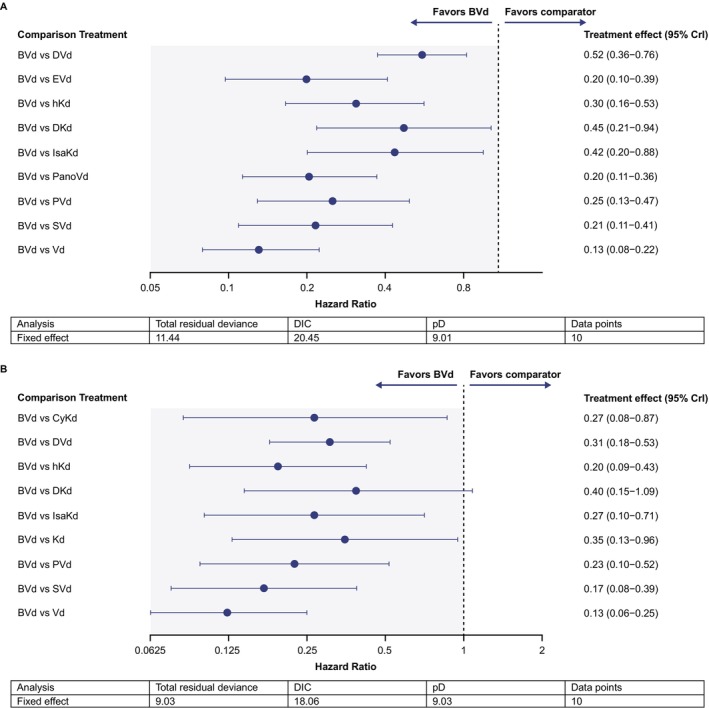
Fixed‐effect BVd treatment comparisons for 1 prior line (A) and high‐risk cytogenetic populations (B) by PFS. BVd, belantamab mafodotin + bortezomib + dexamethasone; CrI, credible interval; CyKd, cyclophosphamide + carfilzomib + dexamethasone; DIC, deviance information criterion; DKd, daratumumab + carfilzomib + dexamethasone; DVd, daratumumab + bortezomib + dexamethasone; EVd, elotuzumab + bortezomib + dexamethasone; hKd, high‐dose carfilzomib + dexamethasone; IsaKd, isatuximab + carfilzomib + dexamethasone; Kd, carfilzomib + dexamethasone; PanoVd, panobinostat + bortezomib + dexamethasone; pD, effective number of parameters as a measure of model complexity; PFS, progression‐free survival; PVd, pomalidomide + bortezomib + dexamethasone; SVd, selinexor + bortezomib + dexamethasone; Vd, bortezomib + dexamethasone. [Color figure can be viewed at wileyonlinelibrary.com]

A total of 10 studies contributed to the network of evidence for the 1 prior line subpopulation [8, 22, 24, 27, 29, 31, 32, 36, 38, 40]. BVd extended PFS versus all comparators (Figure 3A; HR [95% CrI] range: 0.13 [0.08–0.22] – 0.52 [0.36–0.76]). HRs (95% CrI) reported for comparator regimens of interest versus BVd were 0.45 (0.21–0.94), 0.42 (0.20–0.88), and 0.52 (0.36–0.76) for DKd, IsaKd, and DVd, respectively.

### 3.5 High‐Risk Cytogenetic Subpopulation Outcomes

A total of 10 studies contributed to the network of evidence for the high‐risk cytogenetic subpopulation [8, 22, 25, 27, 32, 36, 37, 38, 39, 49]. BVd improved PFS versus all comparators except DKd, where the CrI for the comparison versus BVd spanned 1 (Figure 3B; HR [95% CrI] range: 0.13 [0.06–0.25] – 0.40 [0.15–1.09]). HRs (95% CrI) reported for comparator regimens of interest versus BVd were 0.40 (0.15–1.09), 0.27 (0.10–0.71), and 0.31 (0.18–0.53) for DKd, IsaKd, and DVd, respectively.

### 3.6 Random‐Effect Models

Results from random‐effects models for the ITT and subgroup analyses were largely consistent with the fixed‐effect models reported here, albeit larger 95% CrIs were reported with the random‐effects models shown in Figures S3‐5.

## 4 Discussion

The head‐to‐head DREAMM‐7 study, which directly compared BVd versus standard of care, DVd, reported a near tripling in median PFS (36.6 months vs. 13.4 months), an early trend toward meaningful OS benefit, and twice as many complete responses or better with BVd compared with DVd (35% vs. 17%) [8]. In the absence of other direct randomized controlled trials (RCTs), this NMA further supports DREAMM‐7 findings, with BVd exhibiting superior benefit versus DVd [8]. This NMA indicated that BVd offered the highest PFS, OS, and ORR of the included PI‐based regimens for RRMM in patients who had received at least 1 prior LOT: BVd improved PFS, OS, and ORR compared with IsaKd and DKd, which are both approved for RRMM treatment [50, 51].

In this study, all results generated with the fixed‐effects model in the lenalidomide‐exposed and ‐refractory populations were in favor of BVd. As lenalidomide is given until disease progression [52, 53] and with frequent use in early LOTs [12], the proportion of lenalidomide‐refractory patients with MM is anticipated to increase. Clinical trials and real‐world studies show that lenalidomide‐refractory patients have inferior PFS and OS compared with non‐refractory patients, highlighting an area of unmet need [13]. Use of lenalidomide in the first and second line, and rising daratumumab use are likely leading to a high number of patients who are triple class‐refractory (i.e., to a PI, an immunomodulatory drug, and an anti‐CD28 monoclonal antibody) [16, 54, 55]. Suboptimal treatment outcomes reported in lenalidomide‐ and daratumumab‐treated patients [13, 56, 57] further demonstrate the need for additional therapies. Assessing the comparative effects of alternative regimens is important to illustrate the potential effectiveness of regimens with different mechanisms of action, such as BVd, in lenalidomide‐refractory patients in early lines, as demonstrated in this NMA.

Analyses in subpopulations who had received 1 prior treatment line highlighted that BVd improved PFS versus all other comparators including DVd, IsaKd, and DKd; these results are particularly notable given that IsaKd and DKd had median PFS values of 35.7 and 28.6 months, respectively, in the ITT populations of the IKEMA and CANDOR studies, with these benefits maintained in patients who received 1 prior LOT [36, 38]. The long median duration of response (95% CI) reported in the DREAMM‐7 study for BVd (35.6 [30.5‐NR] months) versus DVd (17.8 [13.8–23.6] months) highlights the benefit of BVd in early lines with the potential to prolong the time to subsequent therapies [8]. Additionally, patients with high‐risk cytogenetics remain a population with high unmet need who may benefit from the identification of effective treatments [58]. The addition of daratumumab to VRd and Vd resulted in positive outcomes in this patient population [16, 25]. The DREAMM‐7 study showed that BVd was effective in patients with high‐risk cytogenetics (HR 0.36 [95% CI 0.22–0.68] in favor of BVd over DVd) [8]. This NMA demonstrated a PFS benefit with BVd versus other comparator regimens including DVd, DKd, and IsaKd in high‐risk cytogenetic subpopulations.

With increasing treatment options for patients with early RRMM, a need remains for effective treatments in the community setting. Beyond the regimens included in this NMA, chimeric antigen receptor T‐cell (CAR‐T) and bispecific T‐cell engager (BiTE) immunotherapies have shown promising results but have associated risks [59] and CAR‐Ts are associated with additional patient burden and healthcare costs [60, 61, 62].

BVd has the potential to be used in community settings with minimal upfront requirements, and side effects associated with BVd reported in DREAMM‐7 were generally well tolerated and manageable, with anticipated eye‐related side effects such as blurry vision managed through dose and schedule modifications [8].

### 4.1 Strengths and Limitations

Overall, large patient numbers were included in the NMAs; some subgroups had low patient numbers, which may have increased uncertainty in the results. It was unfeasible to consider all outcomes in all populations due to the limitations of reporting in the included studies. A potential confounding factor is the lack of reported patient and clinical characteristics data (e.g., PI refractoriness). Moreover, approximately half of the studies included in the NMA did not report race; therefore, it is not known whether those studies reflect a similar race distribution to other myeloma studies. Additionally, common to studies included in this NMA and more widely, several races/ethnic groups have been underrepresented in MM studies [63, 64] and this could have impacted the reported benefits of various regimens in MM. This NMA did not compare BVd with CAR‐T/BiTes, as no data permitting the connection of CAR‐T/BiTes to the analysis networks were available at the time of the study. Moreover, an indirect comparison of quality of life between regimens was not carried out. Quality of life, role, and physical functioning were reported to be similar between BVd and DVd in DREAMM‐7 [8, 65].

Random‐effects models are used in NMAs to account for between‐study heterogeneity that is not adequately captured by fixed‐effect models. The feasibility assessment for this NMA demonstrated that there was considerable heterogeneity between the studies in the analyses; however, there was only one study per link, which is insufficient to reliably estimate between‐study variance. Therefore, the primary analysis for this NMA used the fixed‐effects model, and the random‐effects model was a secondary analysis. Random‐effects model results were aligned with results from the fixed‐effects model. Additionally, deviance information criterion values and residual deviances were similar across models. Moreover, due to the nature of the network, which was based on the availability of published studies, the strength of the findings is limited by the inclusion of a single study for each of the direct comparisons except for Vd vs. DVd, thereby limiting the robustness of the indirect comparisons. Pending the availability of results of other eligible studies reporting direct comparisons that could be added to the network, this analysis acknowledges the limitations resulting from single study connections in the network.

To our knowledge, this is the first NMA to compare BVd against standard of care therapies and suggests the potential benefit of introducing BVd in earlier lines of RRMM treatment. As more therapies and combination regimens enter the MM treatment landscape, the scope for head‐to‐head RCTs may diminish and become less informative for therapeutic decisions, requiring further NMAs to identify the most effective approach for treating RRMM [66, 67].

## Author Contributions

All authors were involved in manuscript writing, reviewing, and editing and read and approved the final manuscript. Joshua Richter and Nick Ballew contributed to study conception or design and data interpretation. Ajay Nooka, Paula Rodríguez‐Otero, and Fredrik Schjesvold contributed to data interpretation. Emily Combe, Marianne Scott, Leanne Cooper, Indeg Sly, Molly Purser, and Simon McNamara contributed to study conception or design and data acquisition, analysis, and interpretation. Jacopo Bitetti contributed to data analysis and interpretation. Natalie Boytsov contributed to study conception or design and data analysis and interpretation.

## Ethics Statement

The authors have nothing to report.

## Consent

The authors have nothing to report.

## Conflicts of Interest

Joshua Richter reports consultancy fees from Janssen, Bristol Myers Squibb, Pfizer, Karyopharm, Sanofi, Takeda, Genentech, AbbVie, Regeneron and speaker bureau fees from Janssen, Bristol Myers Squibb, Sanofi, Adaptive Biotechnologies. Ajay Nooka has served on advisory boards and received honoraria from Adaptive Biotechnologies, Amgen, AstraZeneca, Bristol Myers Squibb, Cellectar Biosciences, GSK, Janssen, K36 Therapeutics, ONK Therapeutics, Pfizer, Sanofi, Sebia, and Takeda, has received institutional grant/research support from Aduro Biotech, Amgen, Arch Oncology, Bristol Myers Squibb, Cellectis, Genentech, GSK, Janssen, Karyopharm, Kite Pharma, Merck, Pfizer, and Takeda, and has received grant/research support for investigator‐initiated studies from Amgen, GSK, Janssen, Merck, and Takeda. Paula Rodríguez‐Otero reports consultancy fees from Bristol Myers Squibb, AbbVie, Roche, and Pfizer, has received honoraria from Bristol Myers Squibb, Janssen, Sanofi, GSK, Amgen, Regeneron, and Takeda, and is a member on the board of directors/speaker's bureau/advisory committee for Bristol Myers Squibb, Janssen, Sanofi, GSK, Amgen, Regeneron, Takeda, Kite Pharma, AbbVie, Oncopeptides, Pfizer, and GSK. Fredrik Schjesvold has received consultancy fees from AbbVie, Celgene, GSK, Janssen, Oncopeptides, Sanofi, and Takeda, has received research funding from Celgene, GSK, Janssen, Oncopeptides, Targovax, and Sanofi, and has received honoraria from AbbVie, Amgen, Bristol Myers Squibb, Daiichi Sankyo, GSK, Janssen, Novartis, Oncopeptides, Pfizer, Sanofi, SkylineDx, and Takeda. Eirini Katodritou served in a consulting or advisory role for Amgen and Janssen‐Cilag, received travel funding from Genesis Pharma and Takeda, received honoraria from Amgen, Genesis Pharma, Janssen‐Cilag, and Takeda, and received research funding from Amgen, Genesis Pharma, Janssen‐Cilag, and Takeda. Philippe Moreau served in a consulting or advisory role for Amgen, Celgene, GSK, Janssen, and Takeda, and received honoraria from Amgen, Celgene, GSK, Janssen‐Cilag, Novartis, and Takeda. Emily Combe, Marianne Scott, Leanne Cooper, and Indeg Sly are employees of FIECON. Nick Ballew, Jacopo Bitetti, Natalie Boytsov, Molly Purser, and Simon McNamara are employees of, and hold financial equities in, GSK.

## Supporting information


Data S1.

## Data Availability

Data used in the analysis were obtained from resources available in the public domain and previously published reports. Additionally, for their studies, GSK makes available anonymized individual participant data and associated documents from interventional clinical studies that evaluate medicines, upon approval of proposals submitted to: https://www.gsk‐studyregister.com/en.
