# Supplementary material for: Belantamab Mafodotin Plus Proteasome Inhibition Efficacy Versus Comparators in Early Relapsed Myeloma: A Systematic Review and Network Meta‐Analysis
Source: Am J Hematol. 2025 Mar 27;100(6):998–1009. doi: 10.1002/ajh.27661 (PMC12067175; doi:10.1002/ajh.27661)
Supplement: Supplementary file 1 — Data S1. [file AJH-100-998-s001.docx]

Title: Belantamab Mafodotin Plus Proteasome Inhibition Efficacy versus Comparators in Early Relapsed Myeloma: A Systematic Review and Network Meta-analysis

Joshua Richter, MD,^1^ Ajay Nooka MD, MPH,^2^ Paula Rodríguez-Otero, MD, PhD,^3^ Fredrik Schjesvold, MD, PhD,^4^ Eirini Katodritou, MD,^5^ Emily Combe, MSc,^6^ Marianne Scott, PhD,^6^ Leanne Cooper, MSc,^6^ Indeg Sly, MSc,^6^ Nick Ballew, PhD,^7^ Jacopo Bitetti, MD,^8^ Natalie Boytsov, PhD,^7^ Molly Purser, PhD,^7^ Simon McNamara, PhD^9^

^1^Mount Sinai, New York, NY, USA; ^2^Winship Cancer Institute of Emory University, Atlanta, GA, USA; ^3^Cancer Center Clinica Universidad de Navarra, Pamplona, Spain; ^4^Oslo Myeloma Center, Department of Hematology, Oslo University Hospital, Oslo, Norway; ^5^Theagenio Cancer Hospital, Thessaloniki, Greece, ^6^FIECON, London, UK; ^7^GSK, Upper Providence, PA, USA; ^8^GSK, Zug, Switzerland; ^9^GSK, Stevenage, Hertfordshire, UK

# **Supplementary Materials**

**Table S1: Combined Medline and Embase search strategy**

**Table S2: PICOS criteria**

**Table S3: Similarity of patient population characteristics (n=13)**

**Figure S1: PRISMA flow diagram of identified publications (A) and studies included in the NMA (B)**

**Figure S2: Network diagram and matrix of treatment comparison**

**Figure S3: Random-effect BVd treatment comparisons for intent-to-treat population by PFS (A), OS (B) and ORR (C)**

**Figure S4: Random-effect BVd treatment comparisons lenalidomide-exposed subpopulation by PFS (A) and ORR (B) and for lenalidomide-refractory subpopulation by PFS (C) and ORR (D)**

**Figure S5: Random-effect BVd treatment comparisons for 1 prior line (A) and high-risk cytogenetic populations (B) by PFS**

## Results

## Table S1: Combined Medline and Embase search strategy

| **Set#** | **Searched for** | **Results**  **Update 1** | **Results**  **Update 2^c^** |
| --- | --- | --- | --- |
| S1 | TI,AB("multiple myeloma") | 123679^a^ | 133515^a^ |
| S2 | TI,AB,IF(myelom*) | 217556^a^ | 208587^a^ |
| S3 | TI,AB(kahler* NEAR/3 (disease* OR morbus)) | 472^b^ | 473^b^ |
| S4 | S1 OR S2 OR S3 | 217604^a^ | 208862^a^ |
| S5 | TI,AB(relaps* or refract* or recurren* or resist*) | 5326656^a^ | 5692874^a^ |
| S6 | TI,AB("prior treatment" OR "prior treatments" OR "prior therapy" OR "prior therapies" OR (previous* NEAR/1 (treat*))) | 153249^a^ | 163010^a^ |
| S7 | TI,AB("second line" or "2nd line") | 84651^a^ | 91959^a^ |
| S8 | TI,AB("third line" or "3rd line") | 17924^a^ | 19710^a^ |
| S9 | TI,AB("fourth line" or "4th line") | 2229^b^ | 2437^b^ |
| S10 | TI,AB(fail* NEAR/3 ("first line" OR "1st line")) | 7499^a^ | 8092^a^ |
| S11 | S5 OR S6 OR S7 OR S8 OR S9 OR S10 | 5479745^a^ | 5856574^a^ |
| S12 | S4 AND S11 | 47441^a^ | 50643^a^ |
| S13 | TI,AB((clinical NEAR/1 trial*) OR ((doubl* OR treb* OR tripl*) NEAR/1 (blind[*3] OR mask[*3] OR dummy)) OR ((control* OR equivalence OR superiority OR non-inferiority OR noninferiority OR pragmatic OR practical OR quasiexperimental OR quasi-experimental OR experimental OR phase) NEAR/3 (study OR studies OR trial* OR group*)) OR sham OR placebo* OR random* OR RCT) OR EMB.EXACT(“clinical trial” OR “multicenter study” OR "phase 1 clinical trial" OR "phase 2 clinical trial" OR “phase 3 clinical trial” OR “phase 4 clinical trial” OR “double blind procedure” OR “crossover procedure” OR “placebo” OR “control group” OR “prospective study”) OR EMB.EXACT.EXPLODE(“randomization” OR “randomized controlled trial as topic” OR “controlled clinical trial”) OR MESH.EXACT(“Randomized Controlled Trials as Topic” OR “Randomized Controlled Trial” OR “Random Allocation” OR “Double Blind Method” OR “Clinical Trial” OR “Placebos”) OR MESH.EXACT.EXPLODE(“Clinical Trials as Topic”) | 8509314^a^ | 9089386^a^ |
| S14 | EMB.EXACT(“case study” OR “case report” OR “abstract report” OR “letter” OR “note”) OR DTYPE(“Letter” OR “Historical Article” OR “Editorial” OR “Note” OR “Comment” OR "News" OR "Newspaper Article" OR “Review”) OR TI,AB(“case study” or “case studies” OR "case report" OR "case reports") | 15349089^a^ | 16179242^a^ |
| S15 | (S12 AND S13) NOT S14 | 12632^a^ | 13942^a^ |
| S16 | S15 NOT ((exp animal/ or exp invertebrate/ or animal experiment/ or animal model/ or exp plant/ or exp fungus/) not exp human/) | 11745^a^ | 12981^a^ |
| S17 | S16 AND LA(english) | 11493^a^ | 12717^a^ |
| *S18 (Upd 1)*S18 | *S17 AND PD(>20211201)*S17 AND PD (relevant date inserted here) | 740^b^ | --817^b^ |
| *S18 (Upd 2)* | *S17 AND PD(>20230326)* | -- | 817^b^ |

^a^ Duplicates were removed from the search but included in the result count.

^b^ Duplicates were removed from the search and from the result count.

^c^ Includes combined search from two time points as per the report. Consolidated search reported in this table for Update 2 was recreated to represent the total search time horizon.

## Table S2: PICOS criteria

| **PICOS elements** | **Criteria** |
| --- | --- |
| Population | Adults (aged ≥18 years) with documented MM, previously treated with at least one prior LOT, and with documented disease progression during or after most recent therapy |
| Intervention | Any treatment or combination of treatments, including but not restricted to:   - **Anti-BCMA ADC therapies:** Belantamab mafodotin (GSK 916) and other ADC therapy - **Proteasome inhibitors:** Bortezomib, carfilzomib, ixazomib and other PIs - **Immunomodulatory drugs:** Lenalidomide, pomalidomide, thalidomide and other immunomodulatory drugs - **Corticosteroids:** Dexamethasone and others - **Alkylating agents:** Cyclophosphamide, cisplatin, melphalan, bendamustin and others - **Peptide-drug conjugates:** Melphalan flufenamide and others - **Other chemotherapeutic agents:** e.g., doxorubicin, etoposide and others - **HDAC inhibitors:** Panobinostat and others - **Anti-CD38 therapies:** Daratumumab, Isatuximab and others - **Anti-SLAMF7 therapies (CS1/CD319/CRACC):** Elotuzumab - **Exportin1 (chromosome region maintenance 1) antagonists:** Selinexor - **Programmed cell death protein 1 (PD-1)/ Programmed death ligand 1(PD-L1) inhibitors:** Pembrolizumab, nivolumab and others - **Anti-CTLA4:** Ipilimumab - **Anti-APRIL therapies:** BION-1301 - **BcL-2 inhibitors:** Venetoclax - **eEF1A2 antagonists**: Plitidepsin - **VEGFR inhibitors:** Vatalanib - **HSP90 inhibitors:** Tanespimycin - **Hypomethylating agents:** Azacytidine - **Anti-BCMA CAR-T-cell therapies:** Idecabtagene vicleucel (Ide-cel) - **T-cell therapies:** Elotuzumab bispecific antibody, ciltacabtagene autoleucel (cilta-cel), REGN-5458, CC-93269, letetresgene autoleucel (lete-cel) - **Anti-BCMA CD3/bispecific antibody therapies:** Elranatamab (PF-06863135), teclistamab (JNJ-64007957), talquetamab (JNJ-64407564) AMG 420, AMG 701, TNB-383B, Descartes-08 - **Cereblon E3 ligase modifiers (CELMoD):** CC-92480, Iberdomide (CC-220) - **Bromodomain and extra-terminal inhibitors:** RO6870810 - **Radiopharmaceuticals:** CLR-131 and others   With the exception of:   - Surgery - Palliative treatment - Radiotherapy - Autologous stem cell transplant (ASCT) alone |
| Comparators | - Between above intervention comparisons - Standard of care/ best supportive care - Placebo or no treatment - No dose finding/single intervention comparisons |
| Outcomes | **Studies reporting efficacy outcomes of interest of the 2L+ RMMM population, including but not restricted to:**  OS, PFS, PFS2, CR, sCR, PR, VGPR, MR, SD, PD, PPS, MRD negativity, ORR, DoR, TTBR, TTR, TTP, TTTF, TTNT  **Safety outcomes, including but not restricted to:**   - Total AEs greater than 5%: Hematologic AEs (total), Non-hematologic AEs (total), Grade 3+ AEs, total TRAEs, grade 3+ TRAEs, total SAEs (≥5%), discontinuations due to AEs, time to treatment discontinuation, treatment-related deaths - Target AE’s (regardless of % reported): The following AEs will be included (total and grade 3+): anaemia, constipation, CRS, diarrhoea, dyspnoea, fatigue, febrile neutropenia, HLH/MAS, ICANS, neutropenia, ocular toxicity, pneumonia, pyrexia, thrombocytopenia, URTI, hepatic toxicity, neurotoxicity, leukopenia |
| Study design | Primary and post hoc analyses of:   - RCTs |
| Publication Type | - Full-text peer-reviewed articles (i.e., no narrative reviews, editorials, protocols, letters, notes or comments) - Clinical trial records - Conference abstracts - Relevant GSK clinical study reports, if available |
| Language | - English language only |
| Timeframe | - 2008 to December 2021 |
| Other restrictions e.g., country | - No restriction |

2L+, second-line or later; ADC, antibody-drug conjugate; AE, adverse event; APRIL, A proliferation-inducing ligand; BCMA, B-Cell maturation antigen; BcL-2, B-cell lymphoma 2; CAR, chimeric antigen receptor; CD, cluster of differentiation; CR, complete response; CRS, cytokine release syndrome;
CTLA-4, cytotoxic T-lymphocyte antigen 4; DoR, duration of response; eEF1A2, eukaryotic elongation factor 1 alpha 2; HDAC, histone deacetylase; HLH, hemophagocytic lymphohistiocytosis; HSP90, heat shock protein 90; ICANS, immune effector cell-associated neurotoxicity syndrome; LOT, line of therapy; MAS, macrophage activation syndrome; MM, multiple myeloma; MR, minimal response; MRD, minimal residual disease; ORR, overall response rate; OS, overall survival; PD, progressive disease; PFS, progression-free survival; PFS2, progression-free survival on subsequent line of therapy; PI, proteasome inhibitor; PICOS, Population, Intervention, Comparison, Outcomes and Study Design; PR, partial response; PPS, post-progression survival; RCTs, randomized controlled trials; RMMM, relapsed/refractory multiple myeloma; sCR, stringent complete response; SD, stable disease; SLAMF7, signaling lymphocytic activation molecule family member 7; SAE, serious adverse event; TRAEs, treatment-related adverse events; TTBR, time to best response; TTNT, time to next treatment; TTP, time to progression; TTR, time to response; TTTF, time to treatment failure; URTI, upper respiratory tract infection; VEGFR, vascular endothelial growth factor receptor; VGPR, very good partial response

## Table S3: Similarity of patient population characteristics (n=13)

| **Characteristic** | **Studies reporting characteristic (n/N)** | **Comment on similarity/difference** |
| --- | --- | --- |
| Age in years (median) | 11/13 (Missing: NCT01478048^1^, NCT00813150^2^) | - The mean of the median age across the studies was 65.5 (range 63.0–71.0, IQR 64.0–66.3). - Studies NCT00813150^2^ and NCT01478048^1^ did not report median age, alternatively reporting mean age. These values for both studies were within the range reported for median age in the remaining network. |
| Gender (% male) | 13/13 | - The mean proportion of males across the studies was 54.4% (range 49.0–60.0%). |
| Race | 6/13 (Missing: ARROW^3^, BOSTON^4,5^, CANDOR^6,7^, LEPUS^8^, NCT00813150^2^, OPTIMISMM^9^, GEM_KyCyDex^10^) | - White was the most common race group across the studies, ranging from 64.0% to 89.0%. - Asian was the next most common race, ranging from 11.5% to 33.0%. - ‘Other’ races (outside White, Asian, Black) ranged from 0.4% to 13.9%. - Proportions of patients from each race group were comparable across studies.   - The PANORAMA study consisted of White (n=249; 64%), Asian (n=128; 33%), Black (n=5; 1%) and ‘Other’ patients (n=5; 1%) and reported PFS HR (95%CI) for Caucasian (0.69 [0.55–0.86]), Asian (0.54 [0.38–0.78) and Other patients (0.77 [0.27–2.19])^11^and reported OS HR (95% CI) for White (1.06 [0.84–1.35]), Asian (0.77 [0.54–1.10]), and ‘Other’ patients (0.85– [0.31-2.38]) ^12^.   - The ENDEAVOUR study consisted of White (n=348; 75%), Asian (n=58; 13%) and Black patients (n=8; 2%) and reported PFS HR (95% CI) for White (0.52 [0.42–0.65]), Asian (0.60 [0.31–1.16]) and ‘Other’ patients (0.56 [0.32–0.97])^13^. |
| Time since diagnosis, months | 9/13 (Missing: ARROW^3^, ENDEAVOR^13-15^, , NCT00813150^2^, GEM_KyCyDex^10^) | - In the study ITT populations, time since diagnosis was broadly consistent, ranging from 37.5–51.4 months. |

ITT, intent-to-treat; IQR, interquartile range

## Figure S1: PRISMA flow diagram of identified publications (A) and studies included in the NMA (B)

**A**

**B**


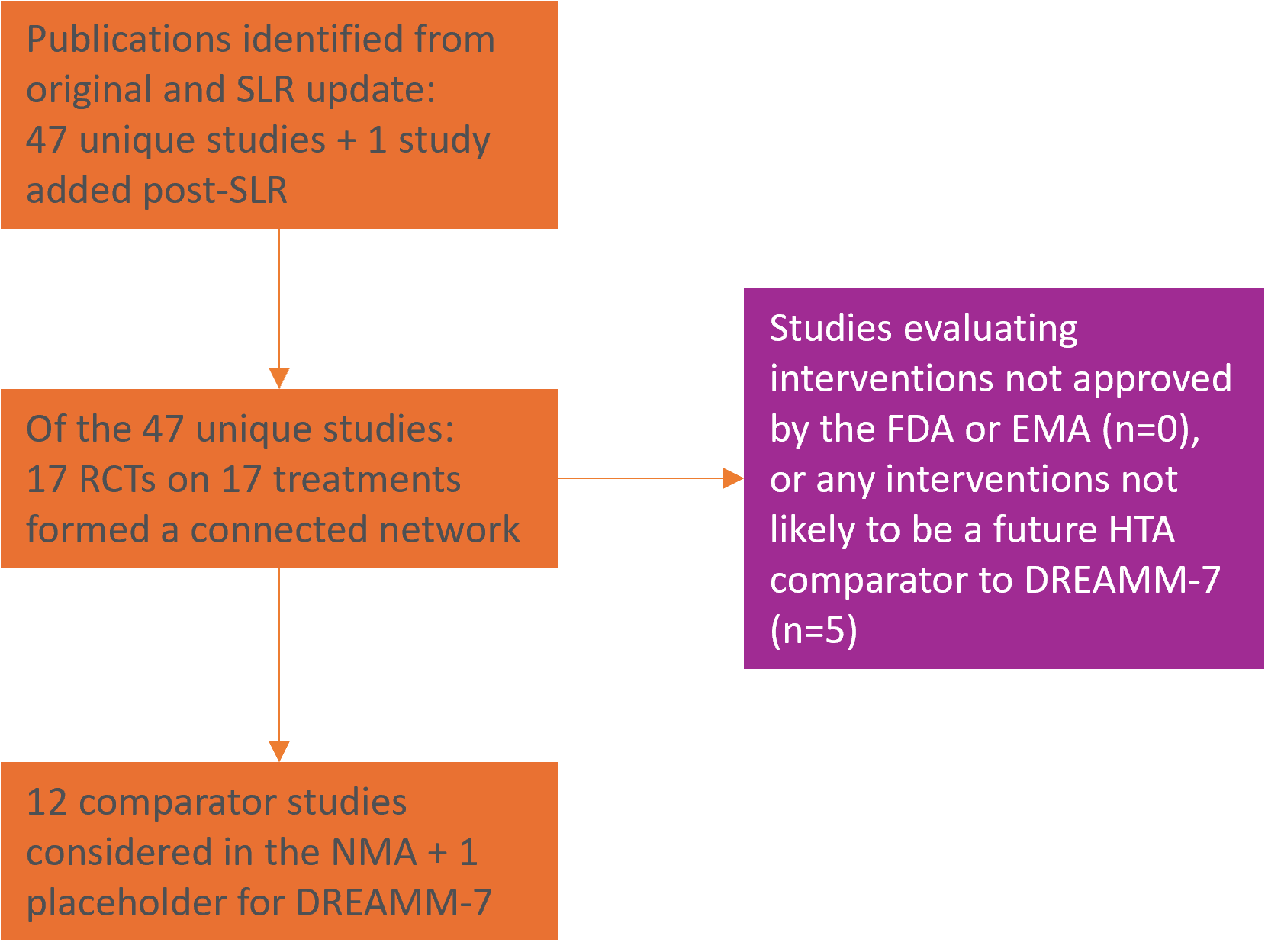


AACR, American Association for Cancer Research; ASH, American Society of Hematology; CDSR, The Cochrane Database of Systematic Reviews; CENTRAL, The Cochrane Central Register of Controlled Trials; DARE, The Database of Abstracts of Reviews of Effects; EHA, European Hematology Association; EMA, European Medicines Agency; HTA, Health Technology Assessment; ICTRP, International Clinical Trials Registry Platform; IMW, International Myeloma Workshop; INAHTA, The International Health Technology Assessment Database; NMA, Network Meta-Analysis; PRISMA, Preferred Reporting Items for Systematic Reviews and Meta-analyses; RCT, randomized controlled trial; SLR, systematic literature review; SOHO, Society of Hematologic Oncology; U.S. FDA, United States Food and Drug Administration.

^a^Not all qualifying studies were included for extraction and studies that were not extracted included treatments and/or trial phases considered lower priority for comparison.

## Figure S2: Network diagram and matrix of treatment comparison


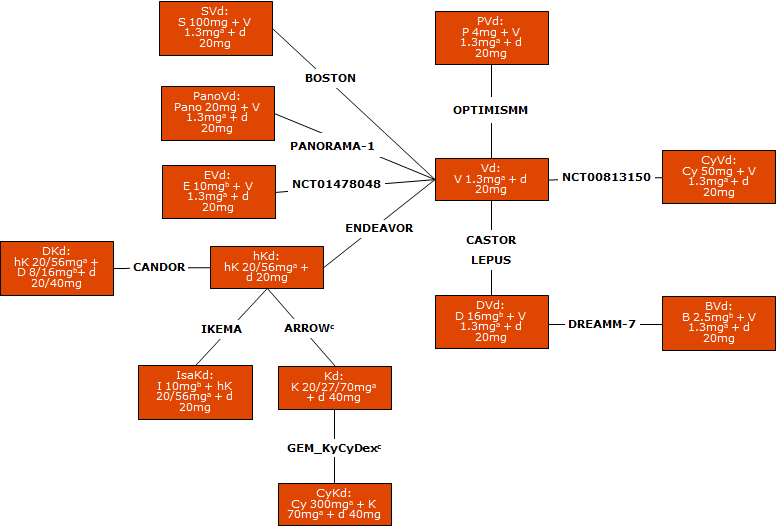


| **Direct comparisons** | **Vd** | **BVd** | **hKd/Kd** ^d^ |
| --- | --- | --- | --- |
| **DVd** | LEPUS, CASTOR | DREAMM-7 | – |
| **PVd** | OPTIMISMM | – | – |
| **SVd** | BOSTON | – | – |
| **PanoVd** | PANORAMA-1 | – | – |
| **CyVd** | NCT00813150 | – | – |
| **EVd** | NCT01478048 | – | – |
| **DKd** | – | – | CANDOR |
| **hKd** | ENDEAVOUR | – | – |
| **Kd** | – | – | ARROW |
| **IsaKd** | – | – | IKEMA |
| **CyKd** | – | – | GEM_KyCyDex |

^a^ mg/m^2^

^b^ mg/kg

^c^ To facilitate inclusion of the ARROW and GEM_KyCyDex studies in the network, equivalence of
the twice-weekly 27 mg/m^2^ carfilzomib and dexamethasone regimen in the ARROW study and the
56 mg/m^2^ carfilzomib and dexamethasone regimen in the ENDEAVOR and CANDOR studies was assumed. This is not expected to materially impact results.

^d^ hKd applies to all studies except GEM_KyCyDex (CyKd vs Kd).

–, no direct comparison; B, belantamab mafodotin; BVd, belantamab mafodotin + bortezomib + dexamethasone; Cy, cyclophosphamide; CyKd, cyclophosphamide + carfilzomib + dexamethasone; CyVd, cyclophosphamide + bortezomib + dexamethasone; D, daratumumab; d, dexamethasone; DKd, daratumumab + carfilzomib + dexamethasone; DVd, daratumumab + bortezomib + dexamethasone; E, elotuzumab; EVd, elotuzumab + bortezomib + dexamethasone; hK, high-dose carfilzomib; hKd, high-dose carfilzomib + dexamethasone; Isa, isatuximab; IsaKd, isatuximab + carfilzomib + dexamethasone; K, carfilzomib; Kd, carfilzomib + dexamethasone; Pano, panobinostat; PanoVd, panobinostat + bortezomib + dexamethasone; P, pomalidomide; PVd, pomalidomide + bortezomib + dexamethasone; S, selinexor; SVd, selinexor + bortezomib + dexamethasone; V, bortezomib; Vd, bortezomib + dexamethasone.

## Figure S3: Random-effect BVd treatment comparisons for intent-to-treat population by PFS (A), OS (B) and ORR (C)

**A**


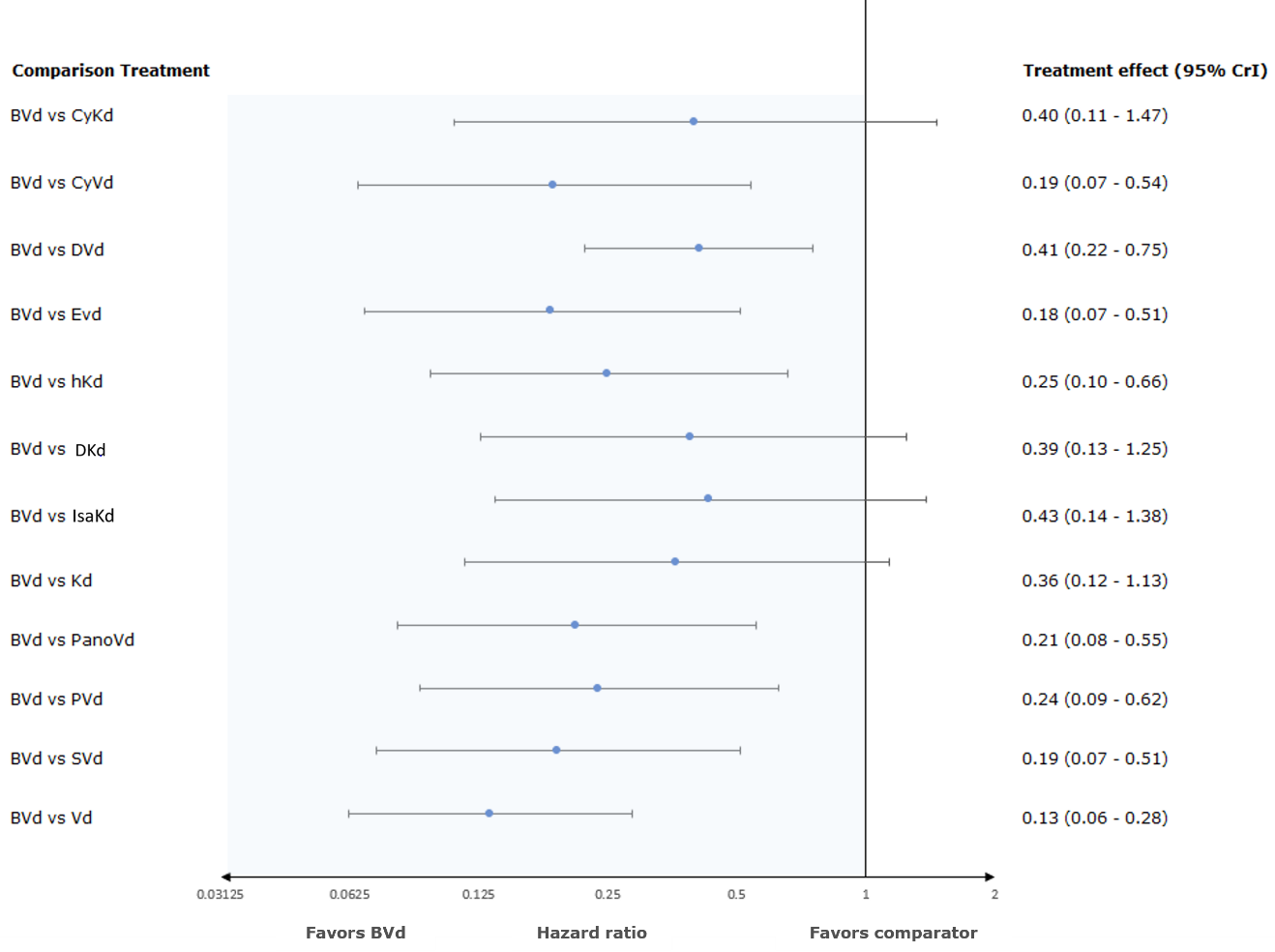


| **Analysis** | **Total residual deviance** | **DIC** | **pD** | **Data points** |
| --- | --- | --- | --- | --- |
| **Random effect** | 12.58 | 25.09 | 12.51 | 13 |

**B**


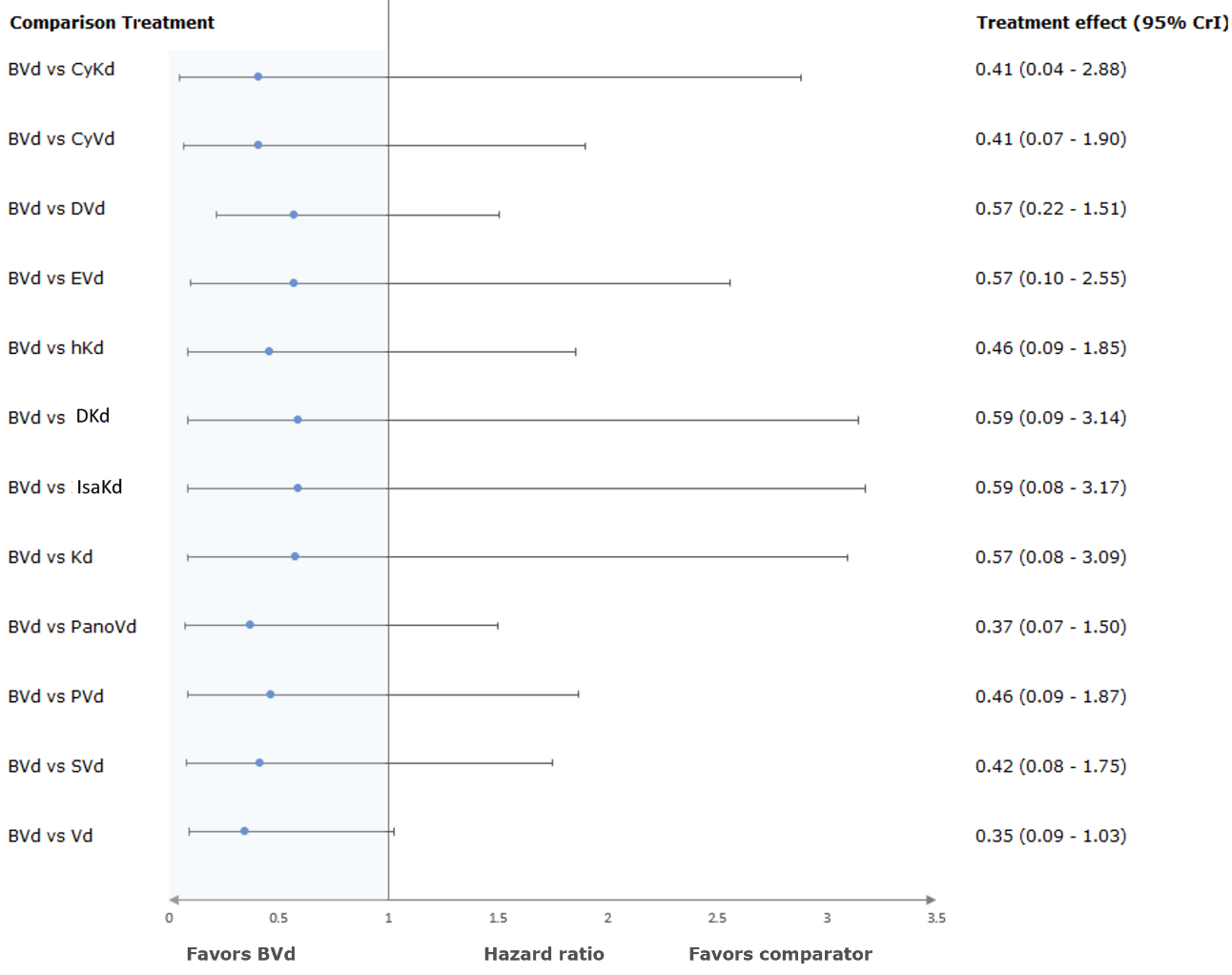


| **Analysis** | **Total residual deviance** | **DIC** | **pD** | **Data points** |
| --- | --- | --- | --- | --- |
| **Random effect** | 13.84 | 26.64 | 12.80 | 13 |

**C**


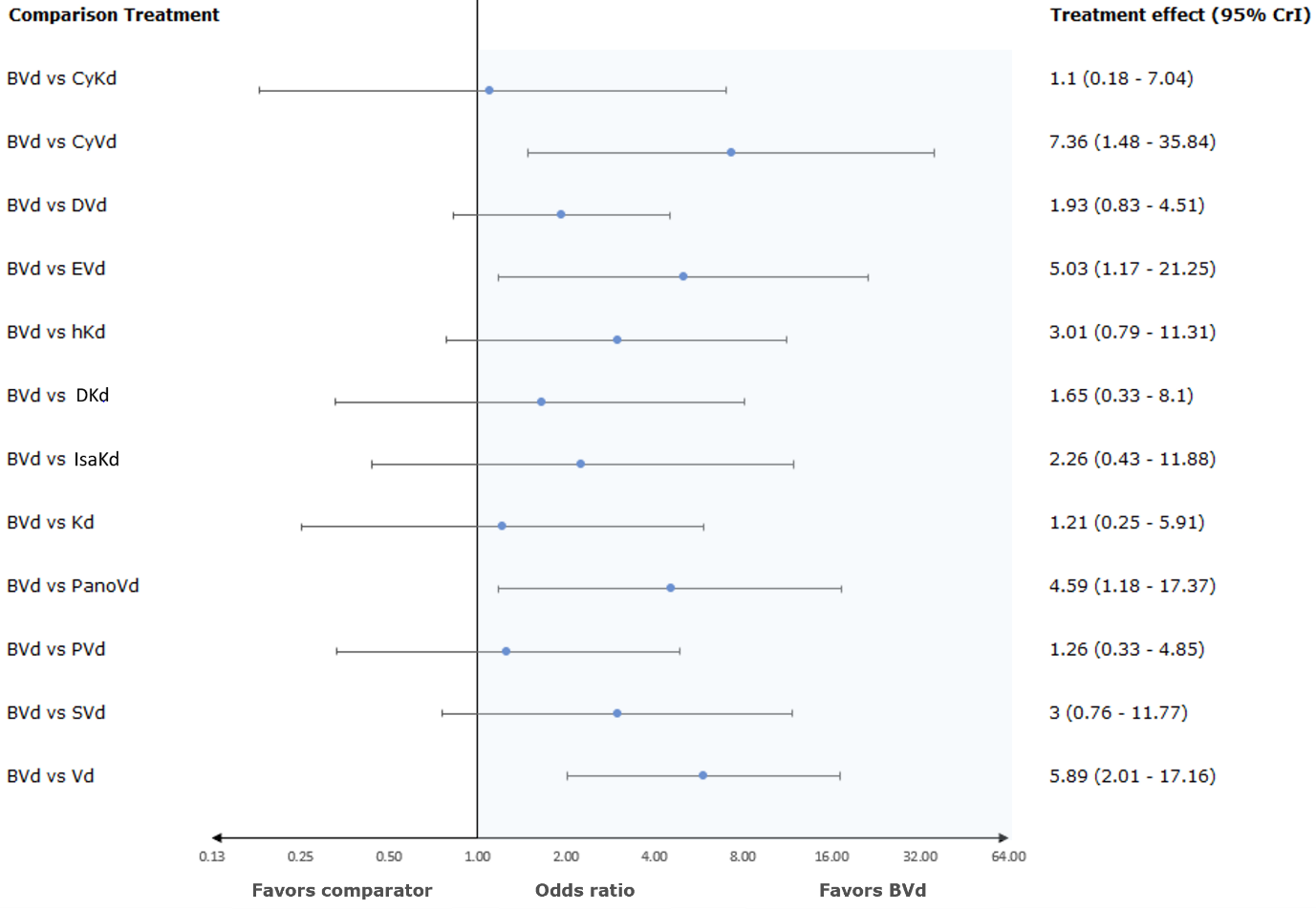


| **Analysis** | **Total residual deviance** | **DIC** | **pD** | **Data points** |
| --- | --- | --- | --- | --- |
| **Random effect** | 25.55 | 51.07 | 25.51 | 26 |

BVd, belantamab mafodotin + bortezomib + dexamethasone; CrI, credible interval; CyKd, cyclophosphamide + carfilzomib + dexamethasone; CyVd, cyclophosphamide + bortezomib + dexamethasone; DIC, deviance information criterion; DKd, daratumumab + carfilzomib + dexamethasone; DVd, daratumumab + bortezomib + dexamethasone; EVd, elotuzumab + bortezomib + dexamethasone; hKd, high-dose carfilzomib + dexamethasone; IsaKd, isatuximab + carfilzomib + dexamethasone; Kd, carfilzomib + dexamethasone; ORR, overall response rate; OS, overall survival; PanoVd, panobinostat + bortezomib + dexamethasone; pD, effective number of parameters as a measure of model complexity; PFS, progression-free survival; PVd, pomalidomide + bortezomib + dexamethasone; SVd, selinexor + bortezomib + dexamethasone; Vd, bortezomib + dexamethasone.

## Figure S4: Random-effect BVd treatment comparisons lenalidomide-exposed subpopulation by PFS (A) and ORR (B) and for lenalidomide-refractory subpopulation by PFS (C) and ORR (D)

**A**


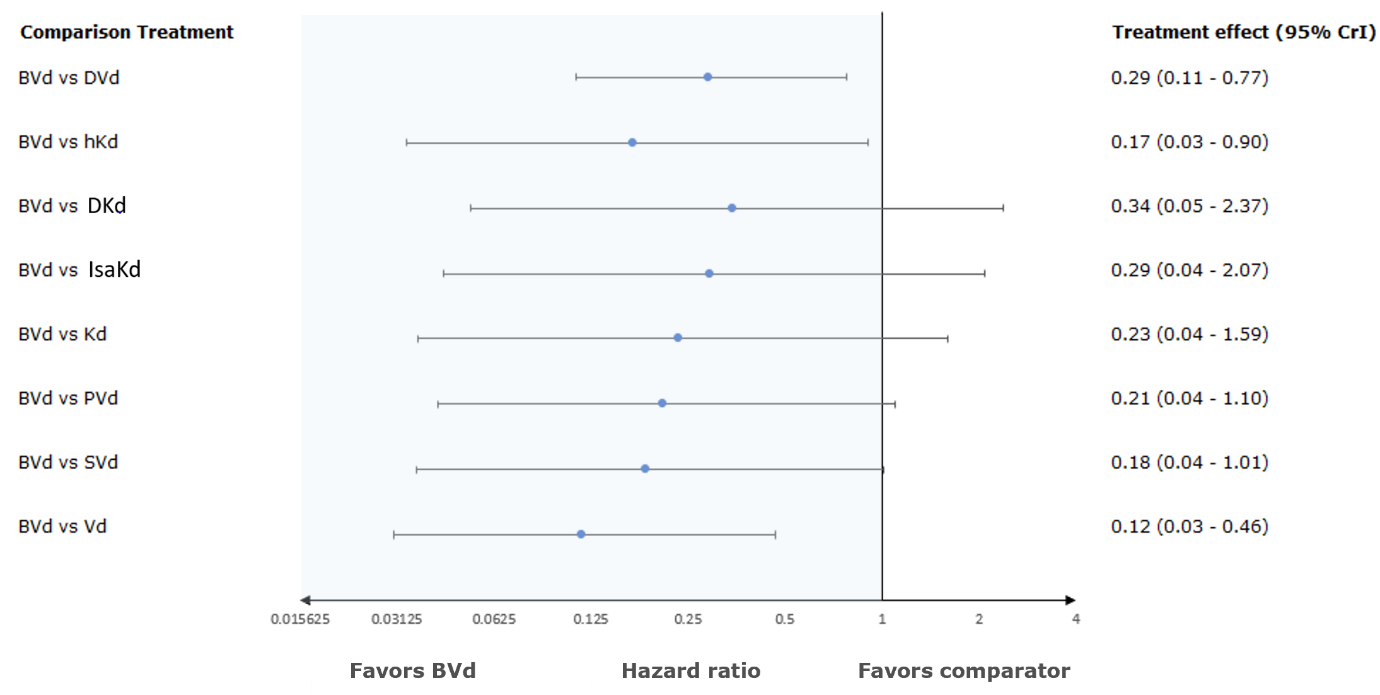


| **Analysis** | **Total residual deviance** | **DIC** | **pD** | **Data points** |
| --- | --- | --- | --- | --- |
| **Random effect** | 8.01 | 16.02 | 8.01 | 8 |

**B**


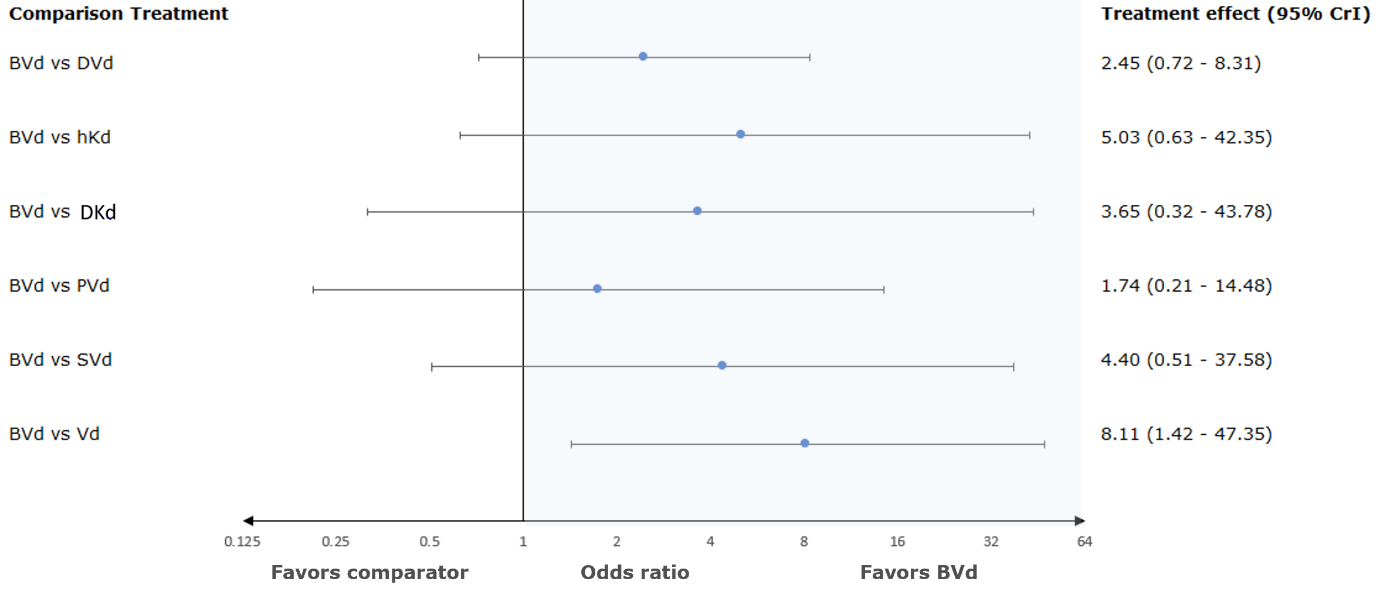


| **Analysis** | **Total residual deviance** | **DIC** | **pD** | **Data points** |
| --- | --- | --- | --- | --- |
| **Random effect** | 12.06 | 24.12 | 12.06 | 12 |

**C**


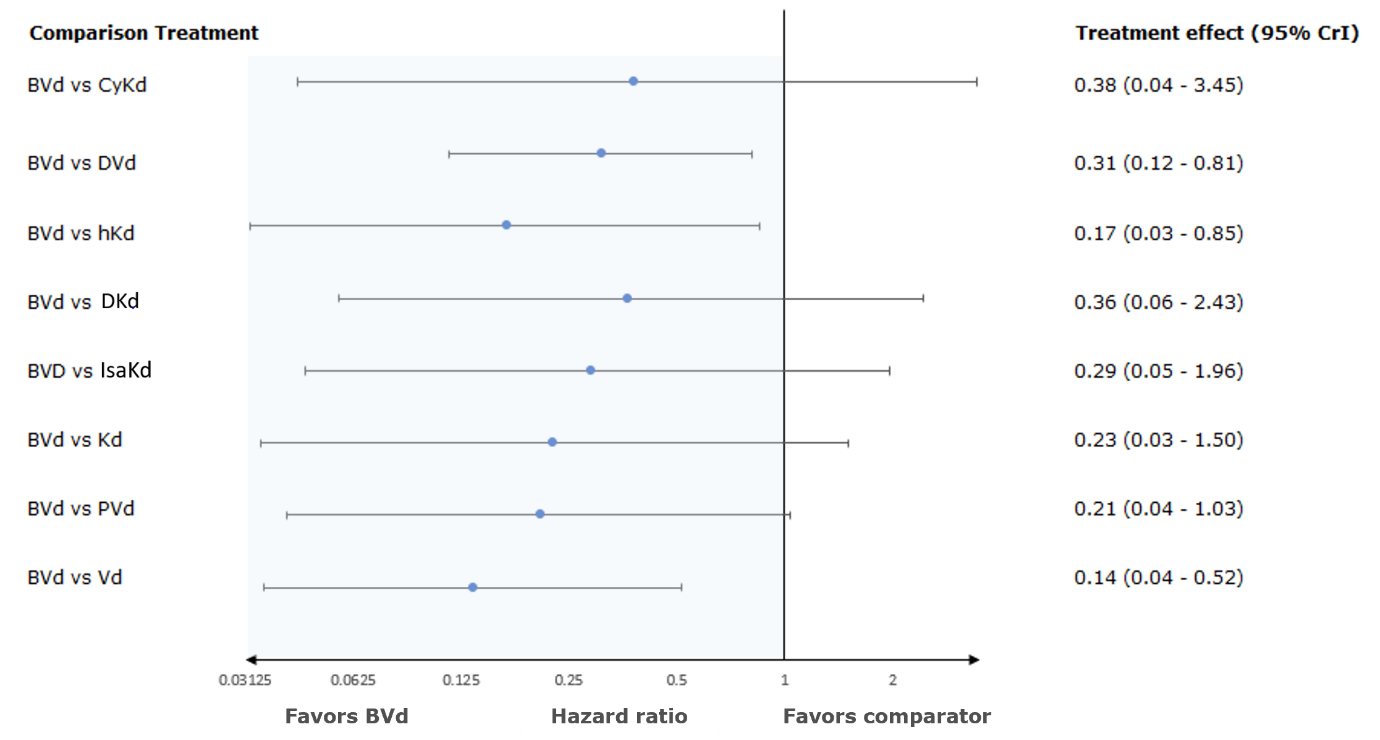


| **Analysis** | **Total residual deviance** | **DIC** | **pD** | **Data points** |
| --- | --- | --- | --- | --- |
| **Random effect** | 8.06 | 16.13 | 8.06 | 8 |

**D**


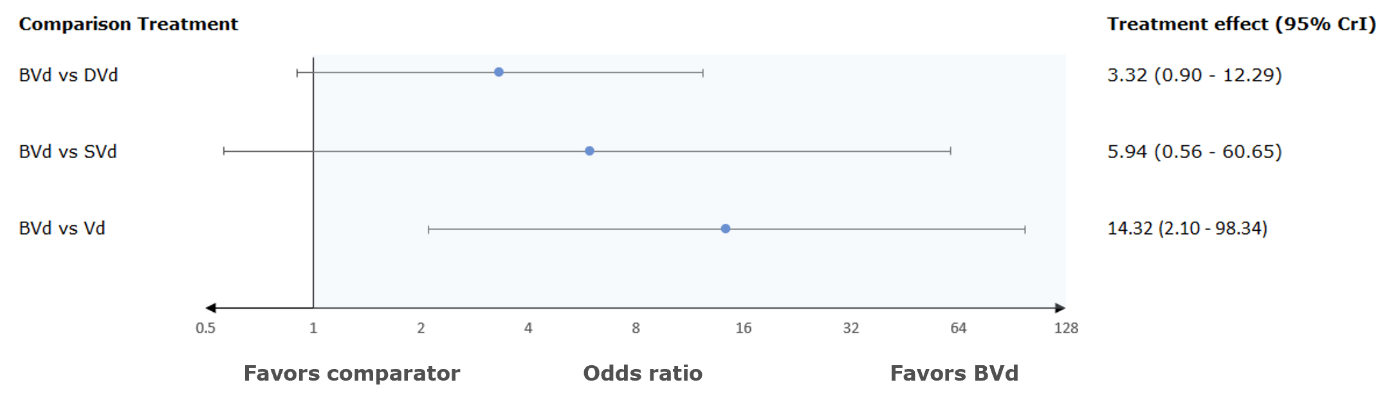


| **Analysis** | **Total residual deviance** | **DIC** | **pD** | **Data points** |
| --- | --- | --- | --- | --- |
| **Random effect** | 6.06 | 12.13 | 6.06 | 6 |

BVd, belantamab mafodotin + bortezomib + dexamethasone; CrI, credible interval; CyKd, cyclophosphamide + carfilzomib + dexamethasone; CyVd, cyclophosphamide + bortezomib + dexamethasone; DIC, deviance information criterion; DKd, daratumumab + carfilzomib + dexamethasone; DVd, daratumumab + bortezomib + dexamethasone; EVd, elotuzumab + bortezomib + dexamethasone; hKd, high-dose carfilzomib + dexamethasone; IsaKd, isatuximab + carfilzomib + dexamethasone; Kd, carfilzomib + dexamethasone; ORR, overall response rate; OS, overall survival; PanoVd, panobinostat + bortezomib + dexamethasone; pD, effective number of parameters as a measure of model complexity; PFS, progression-free survival; PVd, pomalidomide + bortezomib + dexamethasone; SVd, selinexor + bortezomib + dexamethasone; Vd, bortezomib + dexamethasone.

## Figure S5: Random-effect BVd treatment comparisons for 1 prior line (A) and high-risk cytogenetic populations (B) by PFS

A


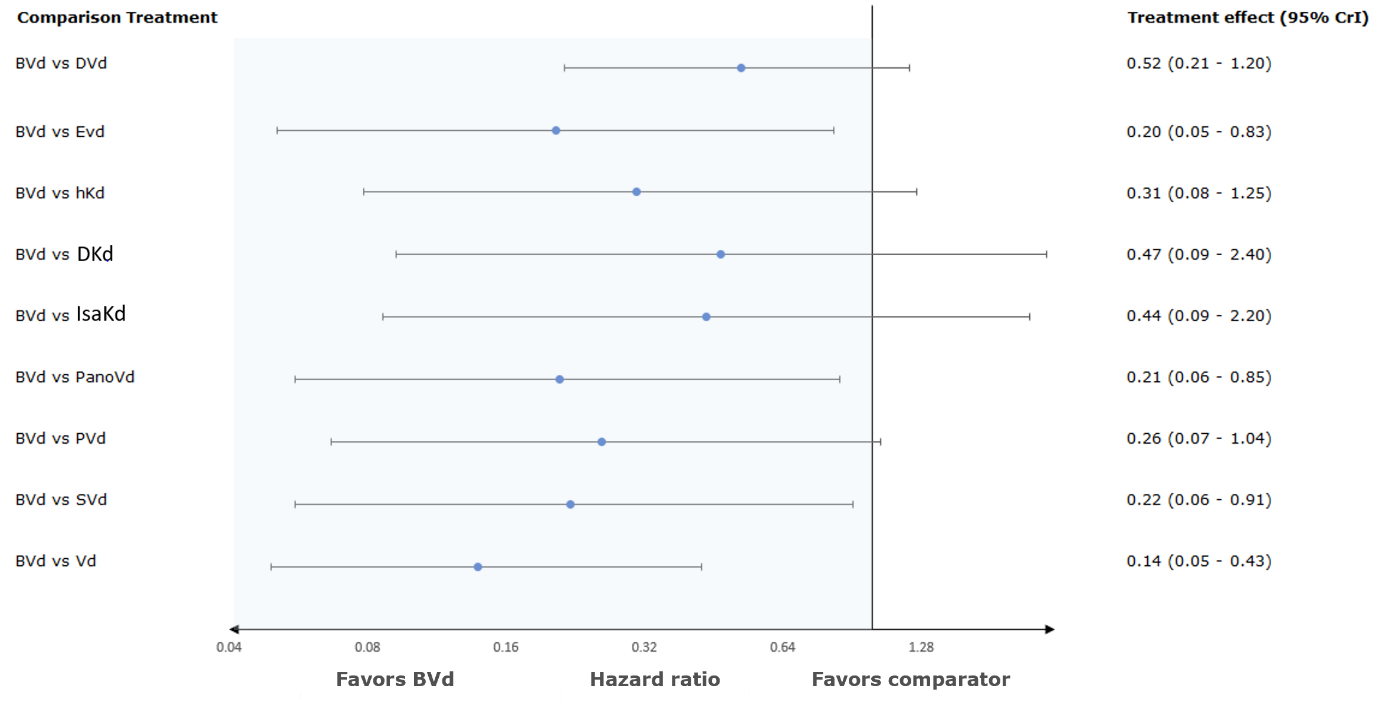


| **Analysis** | **Total residual deviance** | **DIC** | **pD** | **Data points** |
| --- | --- | --- | --- | --- |
| **Random effect** | 10.29 | 19.81 | 9.51 | 10 |

**B**


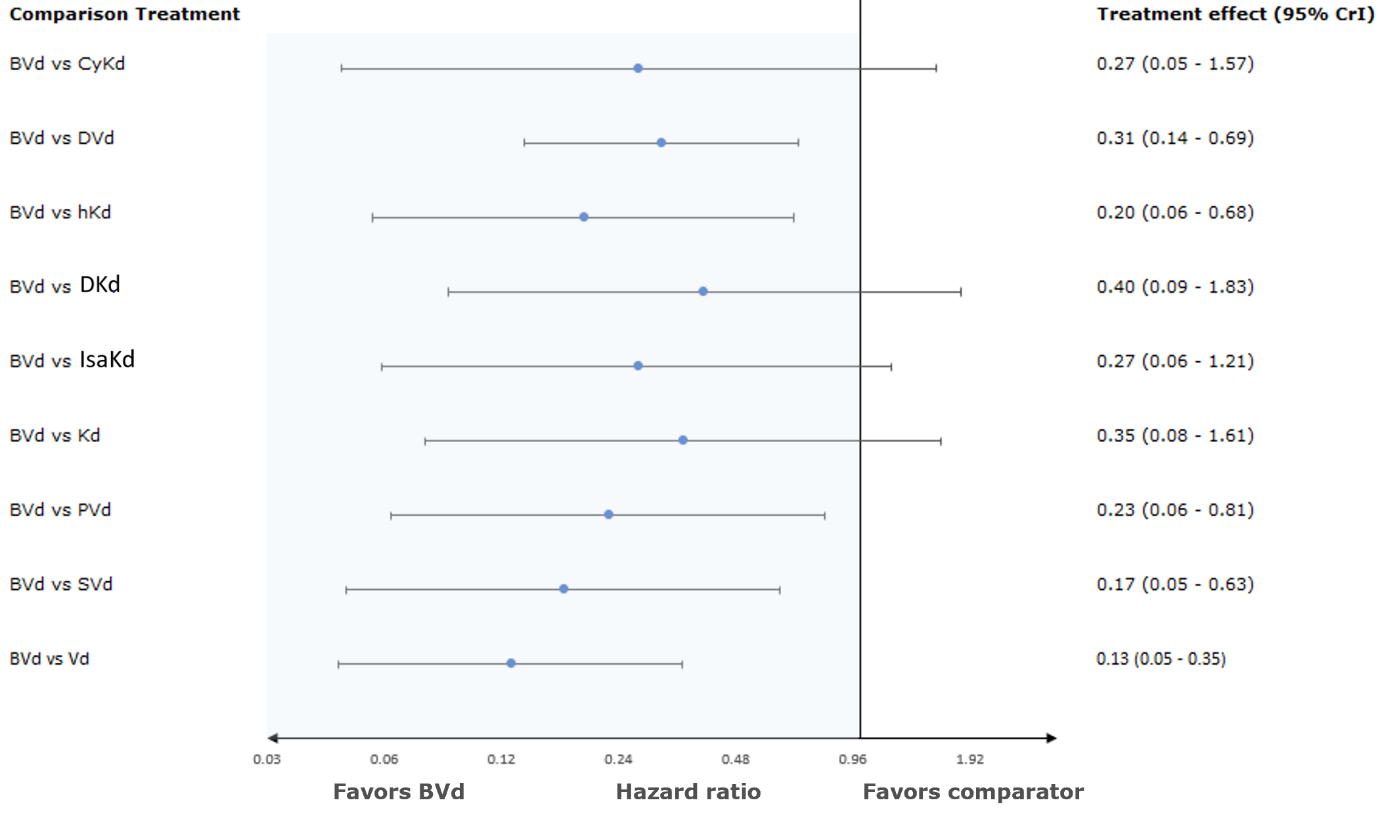


| **Analysis** | **Total residual deviance** | **DIC** | **pD** | **Data points** |
| --- | --- | --- | --- | --- |
| **Random effect** | 9.28 | 18.56 | 9.28 | 10 |

BVd, belantamab mafodotin + bortezomib + dexamethasone; CrI, credible interval; CyKd, cyclophosphamide + carfilzomib + dexamethasone; DIC, deviance information criterion; DKd, daratumumab + carfilzomib + dexamethasone; DVd, daratumumab + bortezomib + dexamethasone; EVd, elotuzumab + bortezomib + dexamethasone; hKd, high-dose carfilzomib + dexamethasone; IsaKd, isatuximab + carfilzomib + dexamethasone; Kd, carfilzomib + dexamethasone; PanoVd, panobinostat + bortezomib + dexamethasone; pD, effective number of parameters as a measure of model complexity; PFS, progression-free survival; PVd, pomalidomide + bortezomib + dexamethasone; SVd, selinexor + bortezomib + dexamethasone; Vd, bortezomib + dexamethasone.

**References**

1. Jakubowiak A, Offidani M, Pégourie B, et al. Randomized phase 2 study: elotuzumab plus bortezomib/dexamethasone vs bortezomib/dexamethasone for relapsed/refractory MM. *Blood.* 2016;127(23):2833-2840.

2. Kropff M, Vogel M, Bisping G, et al. Bortezomib and low-dose dexamethasone with or without continuous low-dose oral cyclophosphamide for primary refractory or relapsed multiple myeloma: a randomized phase III study. *Ann Hematol.* 2017;96(11):1857-1866.

3. Moreau P, Mateos MV, Berenson JR, et al. Once weekly versus twice weekly carfilzomib dosing in patients with relapsed and refractory multiple myeloma (A.R.R.O.W.): interim analysis results of a randomised, phase 3 study. *Lancet Oncol.* 2018;19(7):953-964.

4. Grosicki S, Simonova M, Spicka I, et al. Once-per-week selinexor, bortezomib, and dexamethasone versus twice-per-week bortezomib and dexamethasone in patients with multiple myeloma (BOSTON): a randomised, open-label, phase 3 trial. *Lancet.* 2020;396(10262):1563-1573.

5. Leleu X, Mateos M-V, Jagannath S, et al. Effects of refractory status to lenalidomide on safety and efficacy of selinexor, bortezomib, and dexamethasone (XVd) versus bortezomib and dexamethasone (Vd) in patients with previously treated multiple myeloma. *J Clin Oncol.* 2021;39(15_suppl):8024-8024.

6. Dimopoulos M, Quach H, Mateos MV, et al. Carfilzomib, dexamethasone, and daratumumab versus carfilzomib and dexamethasone for patients with relapsed or refractory multiple myeloma (CANDOR): results from a randomised, multicentre, open-label, phase 3 study. *Lancet.* 2020;396(10245):186-197.

7. Usmani SZ, Quach H, Mateos M-V, et al. Final analysis of carfilzomib, dexamethasone, and daratumumab vs carfilzomib and dexamethasone in the CANDOR study. *Blood Advances.* 2023;7(14):3739-3748.

8. Lu J, Fu W, Li W, et al. Daratumumab, bortezomib, and dexamethasone versus bortezomib and dexamethasone in chinese patients with relapsed or refractory multiple myeloma: phase 3 LEPUS (MMY3009) study. *Clin Lymphoma Myeloma Leuk.* 2021;21(9):e699-e709.

9. Richardson PG, Oriol A, Beksac M, et al. Pomalidomide, bortezomib, and dexamethasone for patients with relapsed or refractory multiple myeloma previously treated with lenalidomide (OPTIMISMM): a randomised, open-label, phase 3 trial. *Lancet Oncol.* 2019;20(6):781-794.

10. Puertas B, González-Calle V, Sureda A, et al. Randomized phase II study of weekly carfilzomib 70 mg/m(2) and dexamethasone with or without cyclophosphamide in relapsed and/or refractory multiple myeloma patients. *Haematologica.* 2023;108(10):2753-2763.

11. San-Miguel JF, Hungria VT, Yoon SS, et al. Panobinostat plus bortezomib and dexamethasone versus placebo plus bortezomib and dexamethasone in patients with relapsed or relapsed and refractory multiple myeloma: a multicentre, randomised, double-blind phase 3 trial. *Lancet Oncol.* 2014;15(11):1195-1206.

12. San-Miguel JF, Hungria VT, Yoon SS, et al. Overall survival of patients with relapsed multiple myeloma treated with panobinostat or placebo plus bortezomib and dexamethasone (the PANORAMA 1 trial): a randomised, placebo-controlled, phase 3 trial. *Lancet Haematol.* 2016;3(11):e506-e515.

13. Dimopoulos MA, Moreau P, Palumbo A, et al. Carfilzomib and dexamethasone versus bortezomib and dexamethasone for patients with relapsed or refractory multiple myeloma (ENDEAVOR): a randomised, phase 3, open-label, multicentre study. *Lancet Oncol.* 2016;17(1):27-38.

14. Orlowski RZ, Moreau P, Niesvizky R, et al. Carfilzomib-dexamethasone versus bortezomib-dexamethasone in relapsed or refractory multiple myeloma: updated overall survival, safety, and subgroups. *Clin Lymphoma Myeloma Leuk.* 2019;19(8):522-530.e521.

15. Siegel DS, Oriol A, Rajnics P, et al. Updated results from ASPIRE and ENDEAVOR, randomized, open-label, multicenter phase 3 studies of carfilzomib in patients (Pts) with relapsed/refractory multiple myeloma (RRMM). *Clin Lymphoma Myeloma Leuk.* 2017;17(1):e142.
